# RETRACTION: Positive Feedback in Cav‐1‐ROS Signalling in PSCs Mediates Metabolic Coupling between PSCs and Tumour Cells

**DOI:** 10.1111/jcmm.70881

**Published:** 2025-10-03

## Abstract

**RETRACTION:** ShaoS. , QinT. , QianW. , YueY. , XiaoY. , LiX. , ZhangD. , WangZ. , MaQ. , and LeiJ. , “Positive Feedback in Cav‐1‐ROS Signalling in PSCs Mediates Metabolic Coupling between PSCs and Tumour Cells,” Journal of Cellular and Molecular Medicine 24, no. 16 (2020): 9397–9408. 10.1111/jcmm.15596.32633891 PMC7417714

The above article, published online on 07 July 2020 in Wiley Online Library (wileyonlinelibrary.com), has been retracted by agreement between the journal Editor‐in‐Chief, Stefan Constantinescu; and John Wiley & Sons Ltd. A third party reported that the BxPC‐3 MCT4 bands in Figure 2G had been duplicated from another article by some of the same authors [Zhang et al. (2016); https://doi.org/10.18632/oncotarget.5677]. Both articles report these bands as originating from different experiments. In addition, the third party reported that the PSC TOMM20 and BxPC‐3 PFKP bands in Figure 2F were duplicated and that the PSC beta‐actin band in Figure 2G had been repeated as the beta‐actin band in Figure 3G and as part of the PSC beta‐actin bands in Figure 4D. An investigation by the publisher confirmed these concerns.

The authors did not respond to an inquiry and request for original data by the publisher. The retraction has been agreed to because the evidence of image re‐use and manipulation both with a previous article and within the same article fundamentally compromises the editors’ confidence in the results presented. The authors did not respond to the notice regarding the retraction.

**RETRACTION:** S. Shao , T. Qin , W. Qian , Y. Yue , Y. Xiao , X. Li , D. Zhang , Z. Wang , Q. Ma , and J. Lei , “Positive Feedback in Cav‐1‐ROS Signalling in PSCs Mediates Metabolic Coupling between PSCs and Tumour Cells,” Journal of Cellular and Molecular Medicine 24, no. 16 (2020): 9397–9408. 10.1111/jcmm.15596.32633891 PMC7417714

The above article, published online on 07 July 2020 in Wiley Online Library (wileyonlinelibrary.com), has been retracted by agreement between the journal Editor‐in‐Chief, Stefan Constantinescu; and John Wiley & Sons Ltd. A third party reported that the BxPC‐3 MCT4 bands in Figure 2G had been duplicated from another article by some of the same authors [Zhang et al. (2016); https://doi.org/10.18632/oncotarget.5677]. Both articles report these bands as originating from different experiments. In addition, the third party reported that the PSC TOMM20 and BxPC‐3 PFKP bands in Figure 2F were duplicated and that the PSC beta‐actin band in Figure 2G had been repeated as the beta‐actin band in Figure 3G and as part of the PSC beta‐actin bands in Figure 4D. An investigation by the publisher confirmed these concerns.

The authors did not respond to an inquiry and request for original data by the publisher. The retraction has been agreed to because the evidence of image re‐use and manipulation both with a previous article and within the same article fundamentally compromises the editors’ confidence in the results presented. The authors did not respond to the notice regarding the retraction.

